# Supporting mental health service users to stop smoking: findings from a process evaluation of the implementation of smokefree policies into two mental health trusts

**DOI:** 10.1186/s12889-020-09673-7

**Published:** 2020-10-28

**Authors:** S. E. Jones, S. Mulrine, H. Clements, S. Hamilton

**Affiliations:** 1 School of Health and Life Sciences, Teesside University, Middlesbrough, UK; 2 School of Geography, Politics and Sociology, Newcastle University, Newcastle, UK; 3 School of Health and Life Sciences, University of the West of Scotland, Ayr, UK

**Keywords:** Process evaluation, Smoking, Mental health, Mental disorder, Tobacco dependence, Psychiatric settings, Smoking cessation, Nicotine dependence, Smoke-free policy

## Abstract

**Background:**

Life expectancy is 10–20 years lower among people with a severe mental health disorder. Most of these early deaths are due to chronic conditions, including cardiovascular and respiratory diseases. Smoking is a major risk factor for these conditions and introducing smokefree policies has been recommended to mental health service providers in England by the National Institute for Health and Care Excellence (NICE), in their Public Health Guideline 48: *Smoking: acute, maternity and mental health services*. This paper reports a process evaluation of introducing these policy recommendations, which were updated in 2013.

**Method:**

Process data were collected through semi-structured interviews with staff (*n = 51*), members of partnering organisations (*n = 5*), service users (*n = 7*) and carers (*n = 2*) between November 2016 – April 2017. Normalization Process Theory (NPT) was used to design the data collection tools and analyse the data. A framework approach was taken with the analysis, using the four concepts of NPT: coherence, cognitive participation, collective action and reflexive monitoring.

**Results:**

The policy made sense to some staff, patients and carers (coherence) who ‘bought-into’ the idea (cognitive participation) but other participants disagreed. Although smokefree policies were operationalised (collective action), sometimes they were opposed. Progress was made, especially in some units, but continued to be resisted in others. Informal appraisal of progress (reflexive monitoring) presented a varied picture.

**Conclusion:**

Some progress has been made in terms of changing an entrenched, smoking culture into one that is smokefree on Trust sites across the region. Perseverance and resourcing over the long-term is required to establish a non-smoking culture in on-site provision of mental health services.

**Supplementary information:**

The online version contains supplementary material available at 10.1186/s12889-020-09673-7.

## Background

Life expectancy is 10–20 years lower among people with a severe mental health (MH) disorder [1]. Most of these early deaths are due to chronic, physical, medical conditions, including cardiovascular and respiratory diseases, for which smoking is a major risk factor [1, 2]. It is now clear that this inequity is not due to increased suicide rates, as previously suggested, but results from socio-economic and health care factors, including smoking behaviour [2]. While smoking prevalence in the general UK population is reducing, it remains high in specific populations, including MH service users [2, 3]. For example: smoking rates among adults with depression are twice as high as among adults without depression; smoking prevalence rates among people with bipolar disorder are significantly higher than in the general population; adults with attention deficit hyperactivity disorder are significantly more likely to smoke than those without and smoking rates among people with schizophrenia are significantly higher than in the general population [4]. As a result, users of MH services, on Trust sites where there is no smokefree policy in operation, are often surrounded by smokers [5].

The smoking culture that exists in MH services and perpetuates smoking dependent behaviours presents a barrier to those who would quit [6]. In addition, the symptoms of nicotine withdrawal are often associated with increased anxiety; consequently, staff can be wary of restricting access to tobacco in the belief that this worsens the person’s mental health condition [7]. Those with mental health conditions tend to be highly dependent smokers, so their withdrawal symptoms are likely to be more marked [5]. This resistance by staff to change their healthcare approaches is linked to several prevailing beliefs held by staff and service users alike, primarily that this population has no desire to quit and will be unsuccessful if they do try [6, 8]. However, some research has shown that these beliefs are unfounded; patients with mental health issues do wish to quit smoking and are able to do so, however, because these individuals often have much higher levels of nicotine dependence, they require more support in order to quit successfully [8, 9].

Resistance appears to come partly from an ethical standpoint, whereby staff feel that it is unfair to deny patients who want to smoke the ability to do so [9, 10]. This is particularly salient when it is believed that the patient uses smoking as a coping mechanism and that removing cigarettes would be detrimental to their mental health, or where the patient considers the facility to be their home [11]. However, where staff believe the policy would actually help patients to quit smoking they tend to view it more favourably [12].

In the UK, there has been a national policy drive to reduce smoking prevalence, mortality and morbidity in the MH population [2, 8, 13–18]. Specifically, Public Health Guideline 48: Smoking: acute, maternity and mental health services, was updated in 2013, and promoted a ‘smokefree’ environment [17]. It recommended preparing and supporting patients to abstain from smoking while in or visiting hospital. Consequently, nicotine management, also known as smokefree, policies were introduced by some mental health service providers [2, 17, 19]. These policies were designed to introduce systems to provide the necessary advice and support, including making available stop smoking advisors and pharmacotherapies, to implement the guideline [17]. These were supported by the Commissioning for Quality and Innovation (CQUIN) framework promoted by NHS England [20], which offers financial incentives to organisations who meet the CQUIN indicators. This paper reports on a process evaluation of the introduction of these policy recommendations in two National Health Service (NHS) Trusts (A and B) providing MH services in an English region. The focus on creating a smokefree environment is only on-site in these Trusts at the time of the study.

This paper adheres to COnsolidated criteria for REporting Qualitative research (COREQ) guidelines to promote transparency [21]. The aim of this paper is to reflect on the process outcomes of the evaluation.

## Method

The theoretical approach used for the process evaluation is Normalization Process Theory (NPT); a mid-range, sociological theory, which has previously been used to understand the issues associated with implementing new policies and practices [22, 23]. NPT has conceptualised the process of bringing in a change into four core constructs: coherence (sense-making), cognitive participation (engagement or buy-in), collective action (activation, doing the work) and reflexive monitoring (appraisal, formal and informal) [22, 23]. Using NPT allowed for reflection on the work required by individuals and organisations to introduce the intervention, how it fitted with current practice (workability) and the modifications necessary for changes to become integrated and embedded i.e. normalized.

There was an extensive planning and preparation period in both Trusts, supported by Public Health England (PHE), prior to implementation of smokefree policies. This included a stakeholder event, the introduction of the Lester Tool to improve routine data collection on physical health [24] and, in Trust A, adoption of the *Preventing ill health by risky behaviours (Tobacco)* CQUIN [20]. PHE were also instrumental in drawing together a steering group for the evaluation, to identify researchers and provide support and guidance during the study. Preparation and the launch for going smokefree in March 2016 had already taken place before the study was approved and data collection began.

### Study design

The process evaluation commenced with a logic model covering inputs/activities/outcomes and impacts (Additional file 1). Interview data were collected from two Trusts, then analysed using a framework approach based on NPT [22, 25].

### Recruitment

All staff employees were eligible to take part. They were primarily invited via adverts circulated within Trusts by the smoking cessation leads via established internal, online communication channels, except for Level 2 Stop Smoking Advisors in Trust B who were recruited via a pre-existing working group. Some staff and the members of partnering organisations were purposively sampled due to their job roles. Staff approached inpatients and separate adverts were sent out via the service user group to outpatients. All service users with mental capacity as designated by the Mental Health Act (MHA) [26] could participate. MHA capacity of inpatients was ascertained from staff; however, it was not queried for the outpatient who was living in the community.

### Sample

Trust A employs over 6500 staff and Trust B employs over 7000. The sample included a variety of staff (*n = 51*), members of partnering organisations (*n = 5*), service users (*n = 7*) and carers (*n = 2*). Any member of Trust staff could answer to the adverts; however mostly clinical staff, with a Trust email account, replied. The sample size was restricted by the response rate. Managers in key positions were purposively sampled and these included both clinical and non-clinical staff. Partnering organisations included the regional Strategic Clinical Network, tobacco control office, patient service user group and both Trusts. Their representatives sat on the project steering group and were interviewed as part of the evaluation.

### Data collection

The four concepts of NPT were used to inform the data collection tools; questions were devised to ascertain if the conceptual stages of normalization were in place during the implementation process (Additional file 2). Coherence is concerned with creating a basis of understanding about the intervention before it is introduced, for example: Why is it necessary? What is its purpose? Cognitive participation involves engaging all stakeholders in thinking about how it might work and preparing for bringing it in. Collective action is when the implementation is put into practice; any gaps in preparation and workability become evident at this stage, as does the degree to which coherence and cognitive participation have been addressed. Reflexive monitoring offers an opportunity to reflect on the implementation process; review and appraise progress, address challenges and promote good practice. The questions aimed to prompt interviewees to focus on these issues.

Interview data were collected from November 2016 – April 2017 through semi-structured individual interviews (16–127 min) and focus groups (around 50 min) by SJ, SM and HC. SJ and SM had prior experience of interviewing for public health research and conducting focus groups. Four staff focus groups were held, two in each Trust. In addition six staff were interviewed by telephone and two staff participants replied by email. Individual interviews were conducted face-to-face, on-site with key staff (Trust A, *n* = 7; Trust B, *n* = 4), except for five who were interviewed by telephone. Representatives from the local tobacco control office and a service user group were interviewed, one face-to-face and one by telephone. A teleconference was conducted with the senior manager in charge of the implementation in each Trust. A focus group was held on-site with service users in Trust A (*n* = 4). Staff were present, but did not participate, while inpatients were interviewed. Three inpatient service users (from Trust B) were interviewed individually face-to-face and two carers (one from each Trust) were interviewed, one on-site and one by telephone. All interviews/focus groups were digitally recorded with the written consent of participant(s).

### Data analysis

Interview data were transcribed and transferred into QSR International’s NVivo 10 Software for qualitative data analysis. Feedback provided via email and any materials used in focus groups (e.g. post-it notes) were also written up and transferred into NVivo 10. Field notes relating to each interview were written up and used as aide memoires during coding but not integrated into the analysis. A framework approach was taken with the analysis [25], to create a way to take complex, qualitative data and systematically input it into a thematic table, then produce findings that could be applied. The five steps in framework analysis are: familiarization, identifying a thematic framework, indexing, charting and mapping/interpretation [25]. Once the analysts had an overview of the data and were familiar with the detail, they created the thematic framework using the four core concepts of NPT; coherence, cognitive participation, collective action and reflexive monitoring were used as a priori themes [22, 23]. Indexing took place as the framework was systematically applied to the data. As NPT is a guiding and not a prescriptive framework, further themes were identified inductively, based on the familiarization and indexing process. The data were then built up into a coherent picture through charting the themes, but drawing on multiple cases, which led to the overall interpretation and findings. Trustworthiness of the findings has four attributes: credibility, dependability, confirmability and transferability. SM and HC met frequently throughout the period of coding to reflect, check for consistency and confirm accuracy of codes and from this a codebook was agreed. SM independently coded interviews, then checked coding with HC, to increase reliability, resolving any discrepancies through discussion. Codes were mapped into themes and subthemes jointly by SM and HC and approved by SJ (Additional file 3). Data saturation was felt to have been reached with regards to staff data, this was determined by the recurrence of descriptions and themes. These methods were used to increase trustworthiness; however, due to challenges in recruitment of patients we acknowledge we were not able to achieve data saturation. Despite this, there was much valuable insight gathered from their inclusion and participation in the research.

## Results

In response to publicising the study, staff, patients and carers volunteered to take part (See Table 1).

**Table 1:** Characteristics of participants

	TRUST A	TRUST B
Key informants (Senior managers)	*n* = 6 Forensic services Pharmacy Estates Project lead Nursing managers (*n* = 2)	*n* = 5 Fire services Pharmacy Estates Project lead Medical manager
Frontline staff	*n* = 13 Level 2 advisors/champions Consultant psychiatrists Nurse practitioners Ward managers Staff nurses Nursing associates Occupational therapists	*n* = 27 Level 2 advisors/champions Consultant psychiatrists Nurse practitioners Ward managers Staff nurses Nursing associates Occupational therapists
Patients	*n* = 4 (male *n* = 1; female *n* = 3; all smokers) Community-based in patient setting	*n* = 1 patient at home (female, smoker) *n* = 2 inpatients (learning difficulties) (both male, smokers)
Carers	*n* = 1 (male, ex-smoker) For inpatient now at home	*n* = 1 (female, ex-smoker) For inpatient now at home
Representatives from partnering organisations on steering group	*n* = 5 Strategic Clinical Network Regional tobacco control office Lead of service user group Medical director Acting medical director	

Key informants were all senior Trust managers. Frontline staff participants included a variety of healthcare professionals. Twenty staff were from Trust A and thirty-one from Trust B; this disparity can be accounted for in the size of the focus groups conducted with Level 2 Advisors (Trust A, *n* = 4; Trust B, *n* = 14). Participants from five organisations, who all sat on the steering group, were interviewed to explore partnership working. These included a member of the Strategic Clinical Network, the local tobacco control office and the service user group, respectively, plus the medical director from one Trust and deputy medical director from the other. Four service users were interviewed in a focus group (3, female; 1, male; all smokers). Interviewed individually were one service user who had been hospitalised previously (female, smoker) and 2 male, inpatient smokers; also one male and one female carer, both of whom were ex-smokers. The service users they cared for were not interviewed.

### Coherence

The findings suggest that some staff were satisfied that introducing smokefree policies was in the patients’ best interest.

*It was quite clear why we were going smokefree, it was to improve the health of the patients, and that patients were dying 15–20 years before the rest of the population, so I felt we had a duty of care for the patients. *

*Frontline Staff, Trust A.*

*This is a NICE public health guideline and as an organisation we had a duty to implement it.*

*Key Informant, Trust A.*

Other staff, although they understood the motivation for smokefree policies, did not agree with the reasoning behind them. In particular, suggesting that they viewed smoking as just one factor amongst others, which contributes to the reduced life expectancy of MH services users.

*I think there’s much more we can do and we can look at in terms of health promotion, in terms of alternatives to prescribing antipsychotics, in terms of polypharmacy, in terms of all those things that we do that have a part to play in people’s life expectancy being so much lower, it just feels like we’ve pinned everything on smoking and I think smoking’s part of a number of processes that are leading to people living less.*

*Key Informant, Trust A.*

Some staff thought it was unethical and contradictory, in terms of insisting patients quit, rather than waiting until they felt ready, as expressed in this focus group:

*… I think part of it is you’re kind of taking the contemplative state away from people – even though we did try to prepare people as much as possible for the smoking ban coming in and did do a lot of work – but still that decision’s kind of being enforced on them.*

*… They haven’t chosen to start with …*

*… and the smoking cessation training again, a huge bit of it is around the contemplation stage.*

*Facilitator: Right that’s interesting isn’t it, so in some ways are you saying the policy doesn’t really …*

*… Reflect smoking cessation advice.*

*Facilitator: Yeah.*

*… Because a big part of it’s about behaviour change and being ready to change, we were trying to get people to stop smoking who weren’t ready to.*

*Frontline staff, Trust B.*

### Cognitive participation

#### Buy-in

Preparation processes were often reported as good, especially regarding hearing experiences of introducing smokefree policies from other Trusts. Although there were mixed levels of buy-in, they were reported to have increased over time in both Trusts. Staff on secure units bought into the policy more than those on non-secure units.

*I think we were so tuned into this coming in and happening that, I would say my practice hasn’t changed because we were already onto this before the policy was ever put in place.*

*Frontline Staff, Trust B.*

For staff involved in the short-term care of patients, they more frequently reported concerns that the policy would not achieve the long-term benefits it purported to.

#### Enrolment

Myth-busting was seen as crucial to buy-in and a central benefit of staff training because pre-existing barriers were reported to enrolment into the changes, including resistance from staff and patients.

*We were aware that there would be some resistance to the stop smoking [policy], so some people were not willing to take it [the champion role] on.*

*Champion, Trust A.*

Applying the policies when a patient was in an acute crisis was often perceived as inappropriate and on occasion actively opposed by staff.

*It is nice to have a detox but it is in the midst of a mental health crisis and they might not be in the frame of mind to deal with that and their smoking addiction.*

*Frontline staff, Trust B.*

Frontline staff identified other barriers in relation to communication and implementation of the policy for a variety of reasons (e.g. senior staff not passing on information, individuals not accessing disseminated information). Ongoing training, to all groups of staff, was seen as an important way to overcome these barriers to uptake, with some stating that evidence of improvement would potentially change the opinions of those resisting the policy.

#### Senior support

Fundamental to buy-in was seeing senior members of the organisation backing the policies; this gave the implementers the authority to act.

*I think the other thing to say is we were, because the way the project was led and it was led by our medical director, there was buy-in from the beginning from senior members of the Trust really so […] our exec management team, everybody had kind of bought in at that level which then fed down, erm kind of through the Trust really*.

*Key Informant, Trust A.*

Where this was not done, there was anecdotal evidence of delays in progression. Critical to success was having a subgroup structure that was prioritised by members, tightly managed and well-focused, with key decision-makers round the table. This enabled implementation of the policies more effectively. Where delegation of responsibility occurred instead, it could lead to further delays, with decisions having to be referred back to the senior manager. Middle management support influenced the outcomes; buy-in at this level was not always translated to the frontline, who felt they lacked the authority to insist on the changes.

*It has to come from senior management. I am new and young, it shouldn’t be on my shoulders … you don’t want to break down the relationships you have with colleagues.*

*Champion, Trust A.*

A lack of consequences for non-compliance by staff was also reported; for example, relating to disciplining staff who smoked in uniform. Enabling access to training was recognised as important in supporting the smokefree message and overall implementation, however this varied between Trusts.

### Collective action

#### Planning

Communication of the reasoning behind the decision to bring in smokefree policies was seen as key in bringing all stakeholders on board. Many participants had found an early stakeholder event useful. Nevertheless, some participants thought that service users, carers and frontline staff were insufficiently consulted during implementation. They expected to be able to discuss the pros and cons of the process and were unhappy with decisions being made at a senior level and handed down rather than co-created.

*Personally I think it would have been better if there was some consultation or at least early on a lot of transparency about why that decision had been made.*

*Partner Organisation Representative.*

Adequate time was thought to have been given to prepare for the going smokefree deadline, although there were a lot of hurdles to overcome to meet it. As well as planned communication strategies, informal communication routes were found to have been instrumental in disseminating the policy to patients and carers. Efforts were made to let patients in the community know about the introduction of the policy, however they were often ill-prepared on admission.

#### Implementation

Certain locations and units were reported as more successful than others in implementing smokefree policies. It was suggested that this was due to the length of stay or security level of the ward and differences in the contexts patients were admitted from e.g. community, prison; and the services available to them prior to admission. However, levels of preparation varied:

*R1: We put in a lot of preparation.*

*R2: We put in very little preparation. It was just the posters went up and the next day, “You’re not smoking anymore”.*

*R1: I don’t know why [specialty] didn’t do what we did. But we made a decision as a directorate very early on.*

*Frontline staff focus group, Trust A.*

*R3: You had it on bulletins and emails and the pharmacy talks. Maybe the preparation of the patients could have been better.*

*R4: I had a different experience, we had a big countdown, then on the day of it, we had a huge healthy living event.*

*Frontline staff focus group, Trust B.*

It was clear that careful use of language was required to encourage smokefree policies to be seen positively. Ambiguity in the policies over patients’ leave compounded any inconsistencies. Consistency of enforcement was another key to success.

*If I was out with a patient and he or she lit up … I would ask them to put it out. And I know for a fact, a good half of the staff members on the ward that I work on, would just say, “Just hide behind that bush and do it [smoke a cigarette] quickly”. We need that continuity …*

*Frontline staff, Trust B.*

Patients’ leave from the ward was seen as a particularly difficult time to manage, when the policy was often likely to be challenged. Participants recognised the smoking culture and some talked about the importance of avoiding the need for enforcement by changing it.

*[There has been a] culture of smoking in mental health. Smoking has been accepted. It has not been seen as problematic.*

*Champion, Trust A*

Visitors entering open sites and smoking in the grounds were a particular challenge. There were many details that needed to be worked out following the introduction of the policies; suggesting a requirement for ongoing review and response in a timely manner.

#### Community links

Communication from healthcare professionals to patients in the community about changes to Trust policy was reported as weak. Although it was recognised that preparing smokers pre-admission was preferable, broken communication channels resulted in staff having to tell patients upon admission that they could not smoke. Similarly, patients admitted from prison reportedly had smuggled in smoking materials. Communication on discharge back into the community was also reported as incomplete, with receipt of messages to healthcare professionals responsible for providing smoking cessation services unclear.

*There’s nothing on discharge yet, we did have a whole referral process in place - a simple form they complete and send it electronic - it’s never been used so we know we’ve got a problem with our staff on the wards who don’t refer*.

*Key Informant, Trust A.*

With variable smoking cessation services on offer in the community, staff expressed a concern that patients would simply be abstaining from smoking as opposed to making a long-term, lifestyle change.

### Reflexive monitoring

#### Positive aspects

There was a view that staff had been more successful in quitting smoking since introducing the smokefree policies. Where the policy was successful, patients’ MH was seen to have improved as they were no longer experiencing nicotine withdrawal symptoms; this had led to a more relaxed atmosphere on the wards, less anxiety in patients, and more time for therapeutic activities.

*Patients are more likely to engage with activities because they want to fill their time; which then progresses onto getting more leave.*

*We have one patient that goes to the gym, one that goes to the library. It [smokefree policy] improves the time they spend off the ward doing meaningful activities.*

*Champions, Trust A.*

In addition, patients felt a sense of achievement following their successful quit attempt, which was reported to improve their mental health.

*I think for some of our patients, because it’s actually a learning disabilities hospital, but obviously a lot of them have mental health issues as well, it increased their confidence and self-esteem. A lot of our patients had poor self-esteem and they actually achieved something by stopping smoking, they achieved something that was extremely difficult and I think it made them think, if we can do that we can do other things as well*.

*Frontline Staff, Trust B.*

#### Negative aspects

Several, unintended, negative consequences of introducing smokefree policies in Trusts were expressed by participants, such as an increase in patients smoking indoors:

*Because we’ve implemented a policy which is driving the smoking underground […] Now we’re having staff having to go into rooms that are filled with smoke and therefore it’s become a second-hand smoking issue*.

*Key Informant, Trust B.*

The hope that reduced smoking would increase patient engagement with activities was not always realised, some used it in other, less active ways:

*We see patients are in bed longer …*

*Frontline staff, Trust A.*

In addition, consequences reported included raised staff stress levels, increased violence and aggression, concerns over ethics and interactions with medication, perceived concerns over the reaction from the external regulator (Care Quality Commission), divergence of opinion between staff and ‘workarounds’ to avoid compliance instigated by patients and staff. It is unclear if these are substantiated by Trust data from alternative sources.

Where patients had informal leave, there were concerns about them smoking off-site or of patients being exploited by local individuals. Although this falls outside the remit of the policies and this evaluation, it is important in terms of holistic care for patients and the impact on-site e.g. it undermines patients’ ability to abstain and staff’s attempts to support them, and it potentially increases difficulty in monitoring antipsychotic drug levels. Staff expressed uncertainty over what was acceptable in nudging patients toward changing their smoking behaviours.

#### Mixed aspects

Whilst in some wards the smokefree policy was introduced relatively easily, in others, staff participants thought there was an increase in challenging or aggressive incidents.

*Where I work, everyone is on board. In forensics a lot of patients will never leave, so there wasn’t a choice.*

*Champion, Trust A.*

*Talking to staff nurses they say violence has increased, and anxiety.*

*Frontline staff, Trust B.*

The therapeutic relationship was reported as being damaged in some cases by the smokefree policies.

*Well it made me feel a bit horrible to one staff because he kept coming out and saying, “Not in here”.*

*Inpatient, Trust A.*

### Enforcement

Enforcement was a key theme that arose organically from the data, it was both a major concern and a signifier of contradictory expectations and practices. For example policies were not always adhered to:

*[There are] still [enforcement] issues as we have staff who disagree very strongly. I suspect some staff are allowing patients to smoke on escorted leave.*

*Frontline staff, Trust A*

Although alcohol was prohibited, some staff viewed smoking differently, and therefore did not think it should be disallowed in similar ways:

*I think drinking alcohol is different because it disinhibits people, and causes violence, so it’s right to prohibit alcohol.*

*Frontline staff, Trust B.*

However others challenged this view, stating buying cigarettes should be treated the same as alcohol:

*Would you report them [a patient on escorted leave] buying a litre bottle of vodka? … you’d report it!*

*Frontline staff, Trust A.*

Staff participants discussed confusion and frustration regarding *how* the policy was to be enforced successfully. Where successful enforcement occurred, it tended to be in settings where patients were used to their behaviours being restricted.

*Working in a forensic setting, I work in an environment where patients are used to not having things and smoking just became one of those.*

*Frontline staff, Trust A.*

Some frontline staff implementing the policy felt that it was at odds with their professional values of ensuring the patients’ best interests.

*I think that we’re affecting choice, we’re just enforcing something that goes against the grain of what we do as nurse.*

*Frontline Staff, Trust A.*

Visitors to the Trust sites, who smoked, also created a challenge to staff implementing smokefree policies. They may be members of the public crossing the site or visitors accompanying outpatients or visiting inpatients. Many of them brought smoking equipment on-site with them.

### Risk

Staff who reported the notion of risk noted that this applied to both staff and patients. Several staff noted concerns about how insisting a patient stop smoking could compromise their own safety (either from aggression or fire). However, some opposite views were also expressed, that there was no noticeable increase in risk from aggression or fire.

*R2: I have also seen the side of, the violence it causes to staff.*

*R1: I have to pick up on that because there has been no increase in violence toward staff since [the smokefree policies came in].*

*R2: Maybe [not] on forensics, but on the adult ward.*

*Frontline staff focus group, Trust A.*

Staff talked about how they felt caught, weighing up the risks between compliance and non-compliance with the policies. Monitoring risk from the interaction between medication and smoking was seen by staff as necessary and concerning but the risk was rarely realised, in their experience. Electronic cigarettes were seen as a potential risk by Trusts, who imposed different and changing restrictions on their use and kept them under review. The wider public were also reported to be at risk e.g. from caches of smoking paraphernalia being found off-site.

### Smoking cessation resources

Policies set out arrangements for provision of NRT to smokers shortly after admission, and were generally adhered to, but there was uncertainty sometimes about access and administration. NRT was not universally accepted by patients as an alternative to smoking, some of whom expressed dislike for NRT products. However, some inpatients who had the opportunity to try different products ahead of the deadline, tended to be more accepting. Smoking cessation behavioural support was reported as variable between Trusts and sites, partly due to challenges in delivering training.

### Patient experience

#### Successful behaviour change

Patients with learning disabilities in secure settings reported quitting successfully, as did a carer, when retelling the experiences of a service user who also quit.

*I smelt them smoking, that’s when it started again. Now that the ban’s in its perfect; saves money as well.*

*Inpatient, Trust B.*

*I didn’t think it would be achievable, but they [staff] managed it [supported my daughter to quit] …*. *So for me it was a bit like a miracle.*

*Carer, Trust B.*

#### Fears and unsuccessful change

Conversely, patients admitted to an acute or informal setting felt pressure and judgement increased but enforcement carried specific challenges.

*… there was people there [Psychiatric Intensive Care Unit (PICU)] who had serious problems who wanted to smoke and they were giving out vaping things but there was no safety net for those who just didn’t want them and they were psychotic …*

*… if they don’t get what they want they really start self-harming.*

*… in the PICU unit it [the smokefree policy] was very well adhered to.*

*Carer, Trust A.*

Quits begun on-site were not seen as well-supported in the community, with patients expecting a negative impact on sustainability. Patients and carers reported that short-term admission was seen as a time of abstinence rather than quitting altogether.

#### Coherence and cognitive participation

Both benefits and concerns were recognised. Overall patients and carers understood the policy and the practical implications. Some patients believed that previously they would have resisted the policy but now realised they had benefitted overall. However, there were doubts expressed by some with regard to the reasoning for going smokefree, as it was still seen by them as a negative experience for patients.

#### Planning and implementation

Positives of stopping smoking with regard to physical health, environmental improvement, social interaction and a personal sense of achievement were expressed by patients/carers. Negatives including psychological stress, impact on social interaction, lack of smoking cessation support in the community and the construction of smoking as deviant were all reported by patients/carers. There were anecdotal successes but also continued resistance to the policies and incidences of smoking by patients off-site.

### Active ingredients

When approaching the data using NPT and logic modelling, active ingredients were identified in relation to implementation of the smokefree policies (Table 2). This analytical process has previously been explored and found to be useful [27].

**Table 2:** Active ingredients identified for normalization

Coherence	Cognitive participation	Collective Action	Reflexive monitoring
Senior support			
Effective leadership			
Prioritisation			
Decision-making sub-groups with sufficient authority			
Inclusive and solution-based approach			
Open communication channels			
Continual resourcing			
Creation of a basis of understanding			
	Legitimisation		
Effective champion(s) operating			
Positive non-smoking discourse			
Thorough preparation			
	Initial and ongoing skills training		
		Perseverance in enacting the policies	
			Ongoing review of systems and processes
			Monitoring smoking-related incidents
			Feedback of ‘wins’ from implementation process to staff and patients

## Discussion

This study aimed to explore the opportunities and challenges of introducing PH 48 [17] into two MH Trusts in England. The process evaluation reported here was designed to capture the attitudes and experiences of staff, patients, carers and partnering organisations during normalization of these changes. In the UK, there is societal support for a smokefree environment as smoking has been banned in enclosed public places, and prevalence overall has been falling [28, 29]. Extending this trend into the MH population necessitates a significant organisational and cultural shift for Trusts [30, 31]. The study logic model set out the hypothesised process for Trusts to become smokefree organisations. The identified inputs, activities and outputs to reach this outcome, were explored with participants.

The study found that implementation of smokefree policies had met with a mixed response. Whilst progress was made in both Trusts, many challenges were also highlighted. This was also reflected in the effectiveness outcomes (reported elsewhere), as measured within the routinely collected data, which remained inconclusive; leading the analyst to recommend that more detailed, high-quality data requires entering onto the patient administration system, to capture the effectiveness of smokefree policies [32].

### Normalization of smokefree policies

#### Coherence and cognitive participation

According to NPT, coherence and cognitive participation are required to progress to action and normalization [22]. There were examples in both Trusts where this foundation had been nurtured, where extensive preparation had taken place and initial implementation progressed better than expected. Nevertheless, these concepts were often reported as lacking. For example: we found that the idea lost coherence for patients when they wanted to quit but lacked the confidence they would succeed. Self-efficacy is known to be a requisite of behaviour change [33] and, without the self-belief in the possibility of change, and lack of confidence in continued support from staff on-site and in the community, they expressed an unwillingness to engage. Similar to Cookson et al. [34], Malone et al. [6] and Mwebe [30], we found that staff, patient and carer perspectives on policy coherence affected their level of buy-in to the implementation, with a persistent belief in pro-smoking ‘myths’. Although systems were being put in place to enable and facilitate harm reduction, many staff were not fully proactive or supportive of the smokefree policies. Misconceptions that were identified, but which are challenged within the literature, included: the ‘right’ to smoke [10, 12], smoking as self-medication, that helps with coping [7], smoking breaks are acceptable [10, 35–37], quitting increases violence [12, 38] and it is not the responsibility of MH staff to support patients to quit [6, 10]. To increase coherence and cognitive participation amongst staff and patients these myths and misconceptions about introducing smokefree policies and quitting smoking require addressing. Training and other forms of communication were reported as being used to do this, with varying degrees of success.

### Promoting normalization through collective action

Implementation science talks about the ‘active ingredients’ of an intervention that bring about the normalization process [39]. These need to be operational for the outcomes to be fully achieved [39]. These active ingredients were operating in some settings and to varying degrees across both Trusts (see Table 2). However, specific threats to normalization reported by participants on the frontline included: lack of consistency of implementation, lack of diversion/alternative activities for patients, lack of staff skills to deal with enforcement and a lack of seamless transition between hospital and community.

### Role of context

It has become increasingly apparent that successfully introducing complex interventions, such as smokefree policies across a region, is highly dependent on context [7, 39, 40]. Different contexts exist between organisations, between sites, between units, between patients and staff [41]. Context goes some way to explain the relative ease and effectiveness when introducing a smokefree environment into learning disability and secure units, compared with units caring for acute and short-term stay patients. For example: it was reported that staff found it easier to prepare the patients and offer continuity where there was lower patient turnover, as in these units. Similarly, in high security units, insisting upon compliance was seen as more consistent with existing practice which included many other restrictions.

### Organisational and cultural shift

It is a decade since Ratschen et al. identified many of the smoking norms in MH facilities that need to change if smoking prevalence is to be reduced; however, according to our findings and other research, many of them remain [8, 10, 30, 35]. Our findings showed that there were genuine fears about the potential for negative consequences, such as increased violence. However, the National Centre for Smoking Cessation and Training is unsympathetic, it says, “The therapeutic management of boredom requires creativity and imagination, facilitating smoking requires neither” [42].

We found that creating cultural change and embedding new policies takes time, personal investment and overcoming resistance to change. Communication of an evidence-based view was found to be essential to bring about this shift – and introducing the active ingredients mentioned above had the potential for integrating and embedding the change e.g. backed by senior management, embedded through staff training, reinforced by an effective champion, well-resourced, with co-created solutions and knowledge exchange between stakeholders.

### Sustainability

Our findings show that, for smokefree policies to be sustainable, they require an extended period to embed and normalize. Requirements included: legitimised and efficient decision-making and communication processes, access to ongoing, good quality training that teaches effective methods, develops a supportive discourse, consistency of enforcement and creates a smooth transition between community/prison and hospital. Reflexive monitoring within NPT demonstrates the importance of keeping the implementation process under review.

#### Strengths and limitations

NPT is one way of understanding the process of implementing and embedding change that is being used increasingly. In this study it was used to focus on the personal experiences of participants. This allowed for an exploration of the attitudes and perspectives of participants, and to understand the opportunities and challenges this raised, when introducing smokefree policies in two MH Trusts. Different media, such as face-to-face, telephone, teleconference and email, were used to collect the data in response to participants’ preferences. This variation in method might have changed the data in terms of how it was expressed or received; however, the researchers were cognisant of this and it offered the opportunity for input from a wider group of people. Issues such as the organisational structures underpinning the workforce, and the management of change, became evident, but were not fully explored. During recruitment, a number of communication methods were used, nevertheless responses from frontline staff, patients and carers were low, compared to the number of employees in the sample. There were probably several reasons for this, however they were not explored within the data. Data saturation was not reached and, primarily, participants were self-selecting, which might have introduced some bias, as more themes may have emerged and those with most interest in the topic may have volunteered. Some issues were raised within the data that required corroboration from other sources which were not within the remit of the project. Participant data and environmental contexts were limited to various sites and departments in two Trusts within an English region and may not reflect the situation in other organisations or locations.

## Conclusions

Implementation science can contribute towards understanding the opportunities and challenges to implementation in complex healthcare settings by using interpretive theories and methodologies, such as NPT and logic modelling. Inroads have been made in terms of changing an entrenched, smoking culture into one that is smokefree on Trust sites; however, there remain variations across specialties and many challenges to full implementation. Once there is sufficient buy-in to a non-smoking culture it is anticipated that the issues relating to enforcement and perceived risk will diminish. Perseverance over the long-term is required to establish smokefree sites in participating MH trusts, supported by robust, routine, quantitative data collection systems.

### Supports what is already known

Smoking rates and levels of dependency are high in psychiatric populations and cessation is more challenging than for the general population. PH 48 [17] argues that introducing a smokefree culture into Trusts offers an opportunity for patient and staff benefit in terms of physical and mental health and is achievable with appropriate support. Nevertheless, there is a longstanding smoking culture in MH care, wherein smoking is seen to be both acceptable and beneficial in some circumstances.

### What does this paper add?

NPT and logic modelling are helpful in increasing understanding of the dynamic implementation process. Using NPT has identified new knowledge in terms of the challenges to implementation that persist at this time with regard to introducing and embedding smokefree policies into two MH Trusts; change is slower than expected, and even with a societal ban, pro-smoking beliefs persist.

## Recommendations

The inconclusive and slow progress made during the implementation highlights the need for further research into the details of normalizing smokefree cultures in MH settings.

Sharing of experience is recommended; examples of success in other Trusts was a powerful way to motivate and inform.

## Supplementary information

Additional file 1. Logic model_Qualitative data. (PPTX 44.6 kb)

Additional file 2. Interview Schedules. (DOCX 24.4 kb)

Additional file 3. Coding framework. (DOCX 17.0 kb)

## Data Availability

No interview data are available to share in their entirety because the ethical approval for the study was on the basis of the research team only having access to the raw data. The executive summary for the study report is available at: http://www.fuse.ac.uk/askfuse/outputs/Evaluation%20of%20the%20introduction%20of%20smokefree%20policies%20in%20two%20North%20East%20NHS%20Foundation%20Trusts%20-%20Executive%20Summary.pdf.

## Abbreviations

COREQ: COnsolidated criteria for REporting Qualitative research
CQUIN: Commissioning for Quality and Innovation
MH: Mental Health
NHS: National Health Service
NPT: Normalization Process Theory
NRT: Nicotine Replacement Therapy
