# Supplementary material for: Supporting mental health service users to stop smoking: findings from a process evaluation of the implementation of smokefree policies into two mental health trusts
Source: BMC Public Health. 2020 Oct 28;20:1619. doi: 10.1186/s12889-020-09673-7 (PMC7594274; doi:10.1186/s12889-020-09673-7)
Supplement: Supplementary file 1 — Logic model_Qualitative data. (PPTX 44.6 kb) [file 12889_2020_9673_MOESM1_ESM.pptx]

## Slide 1
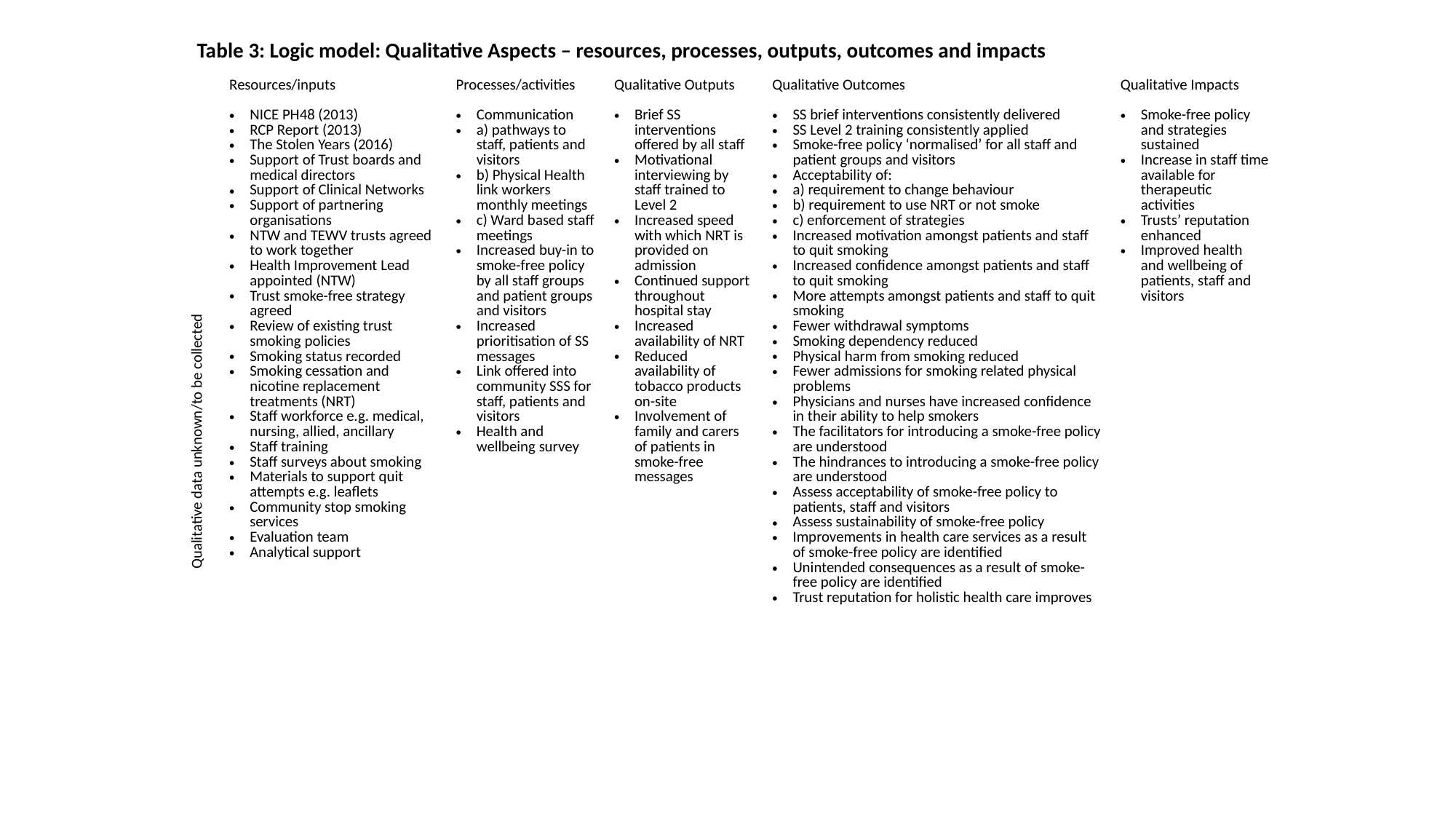

Table 3: Logic model: Qualitative Aspects – resources, processes, outputs, outcomes and impacts
| | Resources/inputs | Processes/activities | Qualitative Outputs | Qualitative Outcomes | Qualitative Impacts |
| --- | --- | --- | --- | --- | --- |
| Qualitative data unknown/to be collected | NICE PH48 (2013) RCP Report (2013) The Stolen Years (2016) Support of Trust boards and medical directors Support of Clinical Networks Support of partnering organisations NTW and TEWV trusts agreed to work together Health Improvement Lead appointed (NTW) Trust smoke-free strategy agreed Review of existing trust smoking policies Smoking status recorded Smoking cessation and nicotine replacement treatments (NRT) Staff workforce e.g. medical, nursing, allied, ancillary Staff training Staff surveys about smoking Materials to support quit attempts e.g. leaflets Community stop smoking services Evaluation team Analytical support | Communication a) pathways to staff, patients and visitors b) Physical Health link workers monthly meetings c) Ward based staff meetings Increased buy-in to smoke-free policy by all staff groups and patient groups and visitors Increased prioritisation of SS messages Link offered into community SSS for staff, patients and visitors Health and wellbeing survey | Brief SS interventions offered by all staff Motivational interviewing by staff trained to Level 2 Increased speed with which NRT is provided on admission Continued support throughout hospital stay Increased availability of NRT Reduced availability of tobacco products on-site Involvement of family and carers of patients in smoke-free messages | SS brief interventions consistently delivered SS Level 2 training consistently applied Smoke-free policy ‘normalised’ for all staff and patient groups and visitors Acceptability of: a) requirement to change behaviour b) requirement to use NRT or not smoke c) enforcement of strategies Increased motivation amongst patients and staff to quit smoking Increased confidence amongst patients and staff to quit smoking More attempts amongst patients and staff to quit smoking Fewer withdrawal symptoms Smoking dependency reduced Physical harm from smoking reduced Fewer admissions for smoking related physical problems Physicians and nurses have increased confidence in their ability to help smokers The facilitators for introducing a smoke-free policy are understood The hindrances to introducing a smoke-free policy are understood Assess acceptability of smoke-free policy to patients, staff and visitors Assess sustainability of smoke-free policy Improvements in health care services as a result of smoke-free policy are identified Unintended consequences as a result of smoke-free policy are identified Trust reputation for holistic health care improves | Smoke-free policy and strategies sustained Increase in staff time available for therapeutic activities Trusts’ reputation enhanced Improved health and wellbeing of patients, staff and visitors |
