# Supplementary material for: Supporting mental health service users to stop smoking: findings from a process evaluation of the implementation of smokefree policies into two mental health trusts
Source: BMC Public Health. 2020 Oct 28;20:1619. doi: 10.1186/s12889-020-09673-7 (PMC7594274; doi:10.1186/s12889-020-09673-7)
Supplement: Supplementary file 2 — Interview Schedules. (DOCX 24.4 kb) [file 12889_2020_9673_MOESM2_ESM.docx]

# **Additional file 2**

## **Interview Schedule (Staff)** Introduce self, check participant has read the information sheet and has had the opportunity to ask any questions, ask for consent to record interview – if not granted explain that you will be taking notes throughout.

Explain purpose of interview – to gain insight into their views on the implementation of smoke-free policy.

What is their role within the trust, and how does it relate to the smoke-free policy?

How long have they worked in that role/for the trust?

Smoker/non-smoker/ex-smoker?

1. Coherence: sense-making - Emphasise this refers to before the policy was implemented.

- What do you understand the basis of the policy to be? What are your thoughts on this?
- Before implementation what measures, if any, were used to raise awareness of the new policy amongst staff? And amongst patients/carers?

Prompt: What did you understand the role of the health improvement lead and other champions to be?

- What outcomes were anticipated to come from implementation of the policy?
  Prompt: How was it expected to influence smoking behaviours of staff and patients within mental health settings? What benefits did the policy aim to achieve? Did you envisage any downsides to the implementation?

1. Cognitive Participation: Engagement

- How was the smoke-free policy received by members of staff?
  Prompt: What were the expectations of staff regarding how the policy would work in practice?
- In what ways, if any, were you able to prepare for the introduction of the smoke-free policy?

Prompt: What, if any, resources were available to staff in order to help them comply with the policy? What, if any, resources were available to staff in order for them to help patients comply with the policy?

- In your experience, how well is the policy understood by other members of staff? And patients?

1. Collective action: putting it into practice

- In what ways, if any, has the policy affected your working practices? And the working practices of others?
  Prompt: Has your workload been altered in anyway? (E.g. more/less time to spend on activities etc.)
- Have staff approaches to encouraging smoking cessation in patients been affected by the policy? If so, please elaborate.
  Prompt: How is Nicotine Replacement Therapy used? What, if any, alternative activities are provided for patients?
- In your experience, how is the policy enforced?

Prompt: Whose role is it to enforce the policy? How do you see your role in enforcing the policy?

- In your experience what support, if any, is available to staff so they can remain smoke-free at work? Would you alter this in anyway?

1. Reflexive monitoring: appraisal

- Do you see any advantages to the policy?

Prompt: Has the policy motivated any changes in behaviour among staff or patients?

- Do you see any disadvantages to the policy?

Prompt: Have patient-staff relationships been affected by the policy in anyway?

- To what extent do you think the smoking behaviour of staff and patients has changed due to the policy?
- To what extent have cultural norms regarding smoking on-site been impacted by the policy?

Prompt: To what extent would you agree that you now have a completely smoke-free working environment?

- In what ways, if any, do you feel the policy has impacted upon your physical health? And that of other staff?
- In what ways, if any, do you feel the policy has impacted upon the physical or mental health of patients?
- Have you identified any outcomes from the implementation of the policy which were unexpected? Could you discuss these?
- What barriers/challenges, if any, do you see to the policy?
- If another trust were intending to go smoke free what would you recommend in order they do so successfully?
- Would you like to see any changes to the policy? If so, what changes would you like to see and why?

Any final comments?

## **Interview Schedule (Patients/Carers)**

Introduce self, check participant has read the information sheet and has had the opportunity to ask any questions, ask for consent to record interview – if not granted explain that you will be taking notes throughout.

Explain purpose of interview – to gain insight into their views on the implementation of smoke-free policy.

Are/were you a patient or carer to a patient who has been affected by the policy?

Gender.

Age.

Ethnicity.

Are you a smoker/non-smoker/ex-smoker? [If carer, also is the patient a smoker/non-smoker/ex-smoker?]

1. Coherence: sense-making - Emphasise this relates to before policy was implemented.

- How were you first made aware of the smoke-free policy?
- What were your initial thoughts on the smoke-free policy?
- What do you understand to be the basis for the trust going smoke-free? What are your thoughts on this?

1. Cognitive participation

- In what ways, if any, were you able to prepare for the introduction of the policy?
- What were you initial feelings towards the policy? How, if at all have these changed over time?

1. Collective action: putting policy into practice

- What influence, if any, do you think the policy has had on patient/staff relationships? Prompt: How has the staff workload been affected by the policy? (E.g. more/less time for activities etc.?)
- What advantages, if any, do you see to the policy?

Prompt: Does it help motivate behaviour change? Is it practical – if so, in what ways?

- What disadvantages, if any, do you see to the policy?

Prompt: Changes to the relationship between patients/between staff and patients? Is it practical – if not, in what ways?

- In your experience how is the policy enforced?

1. Reflexive monitoring: appraisal

- To what extent do you think the smoke-free policy is effective at helping patients/visitors stop smoking?
- To what extent are patients/carers provided with support in order to remain smoke-free, or to quit smoking altogether?
  Prompt: In what ways is Nicotine Replacement Therapy used? What, if any, alternative activities are available to patients?
- What, if any, changes to your physical or mental health have you seen which you believe resulted directly from the policy?
- What barriers/challenges, if any, can you see to the success of the policy?
- Overall, do you feel the policy is effective at creating a smoke-free environment?
- What, if any, changes would you like to see to the policy? Why do you think these would be an improvement?

Any final comments?

## **Service User and Carer Focus Group Schedule**

Derived from the logic model and trust policies

| Timing (minutes) | Stage | Question | Activity |
| --- | --- | --- | --- |
| 0 – 5 | Consent |  |  |
| 5 - 10 | Opening | 1. Each participant introduces themselves and states whether they are a service user (SU) or a carer and their smoking status. | Go round the table asking each participant in turn. |
| 10 - 15 | Introduction / Transition | 2. **Knowledge of the policy**  *Moderator to summarise* | Select short but key elements of the policy and discuss them in small groups. Feedback to whole group about what their extract said and how it was expected to work. |
| 15 - 25 | Key | 3. **Views on the smoke free policy – advantages**  Has introducing the policy helped you in any way?  *Moderator to summarise* | Each SU/carer couple to come up with two positive things about the policy. Write on a post-it and put on chart. |
| 25 - 35 | Key | 4. **Views on the smoke free policy – disadvantages**  *Both:* Has introducing the policy made things worse for you in any way?  *Moderator to summarise* | Each SU/carer couple to come up with two negative things about the policy. Write on a post-it and put on chart. |
| 35 - 40 | Key | 5. **Views on the smoke free policy – level of agreement**  How do you feel about the policy overall?  [Explore understanding of use of cigarettes to cope with life.]  *Moderator to summarise Q3, 4 & 5* | Discuss level of agreement with policy on basis of knowledge of key elements of policy and dis/advantages on post-it notes. |
| 40 - 45 | Key | 6. **Support to reduce harm/quit**  What support do smokers receive?  What support do you think smokers require? | Table discussion |
| 45 - 50 | Key | 7. **Impact on staff workload**  In what ways, if any, do you think the policy has changed staff workloads? E.g. do staff have more time now to do other activities with you? Examples? | Table discussion |
| 50 - 55 | Key | 8. **Enforcement**  How is the smoke free policy being enforced?  Are they appropriate measures?  Are they effective measures? | Present newspaper article on experiences and challenges of hospitals going smoke free in Scotland.  Use this as a basis for a discussion about enforcement. |
| 55 - 60 | Key | 9. **Sustainability**  How effective has the policy been?  What could improve it?  Is it sustainable? | Each SU/carer couple to come up with ways in which the policy could be improved. Write on a post-it and put on chart. |
| 60 - 65 | Ending | 10. **Thank participants** and ask them if there is anything more they would like to add. | Go round the table |

Adapted from Krueger Richard A, Casey Mary Anne. Focus groups: a practical guide for applied research. 5th edition. Los Angeles: Sage, 2015, p47.

## **Frontline Staff Focus Group Schedule**

| Timing (minutes) | Stage | Question | Activity |
| --- | --- | --- | --- |
| 0 – 10 | Consent |  |  |
| 10 - 15 | Opening | 1. **Introduction** – Each participant introduces themselves and states what their role is. | Go round the table asking each participant in turn. |
| 15 - 25 | Introduction / Transition | 2. **Familiarisation with the policy**  - reflection on how the policy was expected to work (logic model)  *Moderator to summarise* | Select short but key elements of the policy and discuss them in small groups. Feedback to whole group about what their extract said and how it was expected to work. |
| 25 - 35 | Key | 3. **How did the policy work in real life scenarios for staff?**  - feasibility, practicality (how is it enforced?)  - effectiveness, impact  - how has it changed your practise?  *Moderator to summarise* | Ask participants to complete a sheet with 2 bullet points under each section. Feedback what they have written. |
| 35 - 45 | Key | 4. **How did the policy work in real life scenarios for patients?**  - practicality  - effectiveness, impact  *Moderator to summarise* | Table Discussion |
| 45 - 50 |  | 5. **What has gone well?**  *Moderator to summarise* | Table discussion |
| 50 - 55 |  | 6. **What would you want the trust to do differently if there was a next time?**  *Moderator to summarise* | Table discussion |
| 55 - 60 | Ending | 7. **Thank participants** and ask them if there is anything more they would like to add. | Go round the table |

Adapted from Krueger Richard A, Casey Mary Anne. Focus groups: a practical guide for applied research. 5th edition. Los Angeles: Sage, 2015, p47.
