# Supplementary material for: Supporting mental health service users to stop smoking: findings from a process evaluation of the implementation of smokefree policies into two mental health trusts
Source: BMC Public Health. 2020 Oct 28;20:1619. doi: 10.1186/s12889-020-09673-7 (PMC7594274; doi:10.1186/s12889-020-09673-7)
Supplement: Supplementary file 3 — Coding framework. (DOCX 17.0 kb) [file 12889_2020_9673_MOESM3_ESM.docx]

**Coding Framework**

| **Coherence** | Reasoning | Understanding of why the Trust made the decision to go smoke-free. |
| --- | --- | --- |
|  | Practical | Understanding regarding what role individuals would have in implementing/upholding the smoke-free policy. |
|  | Professional Values | How does the policy fit with the values of the staff in their professional role? |
| **Cognitive Participation** | Enrolment | What was done to encourage buy-in amongst staff (and patients)? |
|  | Buy-in | How far do staff agree with the policy (and why)? |
|  | Senior Support | Was there initial support from senior staff and has this been sustained? |
| **Collective Action** | Planning | What planning was done in preparation for the implementation? |
|  | Implementation | What work was done to implement the policy? |
|  | Key Individuals | Reference to specific staff members who went above and beyond to make the policy a success. |
|  | Role Impact | How has the policy affected the roles of individuals/wards/professional groups? |
|  | Collaboration | How did groups of professionals work together in planning/implementing the policy? |
|  | Community | What role do the community services play pre-admission and post-discharge? |
| **Reflexive Monitoring** | Positive | What has gone well and why? |
|  | Negative | What has not gone well and why? |
|  | Neutral | Where the policy did not lead to negative outcomes but did not create change. |
|  | Staff | What was the impact of the smoke-free policy on staff (not limited to effects on job role)? |
|  | Patients | What was the impact of the smoke-free policy on patients? |
|  | Trust | What are the implications of the policy to the Trust as a whole? |
|  | Unintended Outcomes | What outcomes, positive or negative, arose from the policy which were not anticipated/intended? |
|  | Recommendations | Direct recommendations made by members of staff to improve/change the policy. |
|  | Future Steps | Where do they see the policy going in the future? How will it progress? |
|  | Staff-Patient Relationship | Has the policy impacted the therapeutic relationship either positively or negatively? |
| **Enforcement** |  | Any reference to enforcement, practically or morally (may fall across more than one Normalisation Process Theory (NPT) component). |
| **Risk** |  | Any mention of risks to staff or patients which are associated with the policy (may fall across more than one NPT component). |
| **Smoking Cessation Resources** |  | What smoking cessation resources were available, how were they intended to be used, how were they actually used, and how effective were they (may fall across NPT components)? |
| **Language/**  **Communication** |  | How is the policy communicated to staff and/or patients in terms of the specific language or terminology? How do staff/patients respond to the terminology (i.e. smoke-free/nicotine management/smoking ban)? |
| **Context** |  | Anything that may be relevant in terms of contextualising findings. |
